# Supplementary material for: In Site Soil Seed-Banks: Size, Composition and Persistence across Tropical Successional Stages
Source: Plants (Basel). 2023 Jul 25;12(15):2760. doi: 10.3390/plants12152760 (PMC10420881; doi:10.3390/plants12152760)
Supplement: Supplementary file 1 [file plants-12-02760-s001.zip › plants-2274660-supplementary.pdf]

S1. List of plant species (≤ 50 cm tall) within different taxonomic families, emerged from the soil seed-bank in primary forests (PF), secondary forests (SF) and old-fields (OF) in Southeast Mexico. Different plant growth-forms are indicated by different letters as follows: EH, epiphytes and hemi-epiphytes; H, herbs; L, woody lianas; P, palms; S, shrubs; and T, trees. Plants emerged directly from the mineral soil in the course of 12 months.

Plant species and/or morphospecies	Family	Successional stage	Growth-form
Abuta panamensis	Menispermaceae	PF	L
Acacia hayesii	Fabaceae	PF	L
Acacia usumacintesis	Fabaceae	PF	T
Acalypha diversifolia	Euphorbiaceae	PF, SF	S
Acanthaceae sp. 1	Acanthaceae	SF	H
Anthurium flexile	Araceae	PF, SF	EH
Anthurium sp. 1	Araceae	PF, SF	EH
Aphelandra deppeana	Acanthaceae	PF	H
Araceae sp. 1	Araceae	PF	H
Bidens pilosa	Asteraceae	OF	H
Bignoniaceae sp. 1	Bignoniaceae	PF	T
Brachiaria decumbens	Poaceae	OF	H
Brosimum alicastrum	Moraceae	PF	T
Calathea inocephala	Marantaceae	PF	H
Calathea macrochlamys	Marantaceae	PF	H
Calliandra sp. 1	Fabaceae	OF	S
Cecropia peltata	Moraceae	PF	T
Celtis iguanaea	Cannabaceae	SF	L
Chamaedorea alternans	Arecaceae	PF	P
Chamissoa altissima	Amaranthaceae	PF	EH
Cissus gossypiifolia	Vitaceae	PF	EH
Cissus sp. 1	Vitaceae	SF	L
Commelina sp. 1	Commelinaceae	PF	S
Asteraceae sp. 1	Asteraceae	OF	H
Conostegia sp. 1	Melastomataceae	OF	S
Cupania glabra	Sapindaceae	OF	T
Curcurbitaceae sp. 1	Curcurbitaceae	SF	EH
Cymbopetalum penduliflorum	Annonaceae	PF	T
Cyperus sp. 1	Cyperaceae	OF	H
Dalbergia sp. 1	Fabaceae	OF	T
Desmodium rastrera	Fabaceae	OF	EH
Desmodium sp. 1	Fabaceae	OF	EH
Doliocarpus dentatus	Dilleniaceae	OF	L

Euphorbiaceae sp. 1	Euphorbiaceae	SF, OF	L
Fabaceae sp. 1	Fabaceae	SF	T
Hamelia patens	Rubiaceae	OF	S
Heliconia sp. 1	Heliconiaceae	SF	H
Ipomoea discolor	Convolvulaceae	OF	L
Iresine arbuscula	Amaranthaceae	PF	T
Loganiaceae sp. 1	Loganiaceae	OF	H
Luehea sp. 1	Malvaceae	SF	T
Lycianthes nitida	Solanaceae	PF	S
Macfadyena uncata	Bignoniaceae	PF	L
Matayba sp. 1	Sapindaceae	OF	T
Mendoncia retusa	Acanthaceae	SF	L
Mikania aromatica	Asteraceae	PF	EH
Mikania sp. 1	Asteraceae	OF	H
Mikania sp. 2	Asteraceae	OF	L
Monstera sp. 1	Araceae	PF	EH
Monstera tuberculata	Araceae	SF	EH
Nectandra salicifolia	Lauraceae	SF	T
Neurolaena sp. 1	Asteraceae	OF	H
Paspalum sp. 1	Poaceae	OF	H
Petridophyta sp. 1	Petridophyta	PF	H
Physalys sp. 1	Solanaceae	OF	H
Piper aequale	Piperaceae	SF	S
Piper hispidum	Piperaceae	SF, OF	S
Piper sp. 1	Piperaceae	SF	S
Polypodium sp. 1	Polypodiaceae	SF	H
Psychotria papantlemsis	Rubiaceae	PF	S
Psychotria chiapensis	Rubiaceae	SF	T
Quararibea funebris	Malvaceae	PF	T
Rhodospatha wendlandii	Araceae	PF	L
Rinorea humilis	Violaceae	SF	T
Rinorea sp. 1	Violaceae	PF	T
Rourea sp. 1	Connaraceae	PF	L
Rourea glabra	Connaraceae	PF	L
Rubiaceae sp. 1	Rubiaceae	OF	H
Schizolobium parahybum	Fabaceae	PF	T
Scleria pterota	Cyperaceae	OF	H
Serjania sp. 1	Sapindaceae	PF	L
Sida rhombifolia	Malvaceae	OF	H
Solanum sp. 1	Solanaceae	SF	L
Spondias radlkoferi	Anacardiaceae	PF	T
Syngonium podophyllum	Araceae	PF, SF	EH
Trichilia erythrocarpa	Meliaceae	SF	T

Trophis racemosa	Moraceae	SF	T
Verbenaceae sp. 1	Verbenaceae	OF	H
Vernonia patens	Asteraceae	OF	S
Vismia camparaguey	Hypericaceae	OF	T
EH sp. 1	Undetermined	OF	EH
Herb sp. 1	Undetermined	SF	H
Herb sp. 2	Undetermined	SF, OF	H
Herb sp. 3	Undetermined	SF, OF	H
Herb sp. 4	Undetermined	SF, OF	H
Herb sp. 5	Undetermined	OF	H
Herb sp. 6	Undetermined	OF	H
Liana sp. 1	Undetermined	SF	L
Liana sp. 2	Undetermined	SF	L
Liana sp. 3	Undetermined	SF, OF	L
Liana sp. 4	Undetermined	OF	L
Liana sp. 5	Undetermined	OF	L
Shrub sp.1	Undetermined	SF	S
Tree sp. 1	Undetermined	SF	T
Tree sp. 2	Undetermined	PF	T
